# Fecal bile acid profiles before and after fecal microbial transplant in pediatric onset ulcerative colitis

**DOI:** 10.1080/29933935.2024.2393219

**Published:** 2024-09-27

**Authors:** Kathleen Lo, Patil Kavarian, Beibei Wang, Riddhi Parsana, Ramon Durazo-Arvizu, Fengzhu Sun, Sonia Michail

**Affiliations:** a Keck School of Medicine, University of Southern California, Los Angeles, CA, USA; b Gastroenterology, Children’s Hospital Los Angeles, Los Angeles, CA, USA; c Pediatric Gastroenterology, Stanford University, Palo Alto, CA, USA; d School of Mathematics, Shandong University, Jinan, China; e Frontier Science Center for Nonlinear Expectations, Ministry of Education, Qingdao, China; f Research Center for Mathematics and Interdisciplinary Sciences, Shandong University, Qingdao, China; g Quantitative and Computational Biology Department, University of Southern California, Los Angeles, CA, USA

**Keywords:** Inflammatory bowel disease, bile acid metabolism, gut metabolites, Hispanic, clostridium difficile

## Abstract

Fecal bile acids (BAs) are key metabolites altered in patients with inflammatory bowel disease (IBD), therefore serving as potential targets of fecal microbial transplant (FMT). To compare changes in fecal BA composition and corresponding microbial transformation pathways in pediatric ulcerative colitis (UC) patients before and after FMT for up to 48 weeks. Fecal BAs, as well as enzymes and bacteria related to BA metabolism were measured in 28 healthy children, and 48 children with mild to moderate UC before and after FMT. Several primary BAs were higher in UC patients at baseline, and subsequently decreased over the 48 weeks following FMT. In particular, the primary BA cholic acid (CA) was higher in UC children at baseline (11.73 pg/mg) compared to healthy controls (8.47 pg/mg), decreased to 10.82 pg/mg at 4 weeks post FMT (p = 0.001) then 10.07 pg/mg at 48 weeks (p = 0.077). Following FMT, the ratio of secondary to primary BAs became more similar to healthy children. The genes coding for bile salt hydrolase, 7α/β-hydroxysteroid dehydrogenase, and bile acid induced operon enzymes were lower in UC patients at baseline, with the majority of them increasing following FMT. Similarly, many of the bacterial genera involved in bile acid metabolism had corresponding increases after FMT.

## Introduction

Ulcerative colitis (UC) is a type of inflammatory bowel disease (IBD) that is characterized by chronic, idiopathic inflammation of the colon. The pathogenesis of IBD is thought to be multifactorial, influenced by genetic predisposition, environmental factors, immunological abnormalities, and altered gut microbiota.^1^ Inflammation from UC causes injury to the intestinal mucosa and leads to poor absorption, affecting the growth and development of children.^2^ The current proportion of pediatric-onset IBD (age <17 at time of diagnosis) is estimated to be 8%.^3^ Incidence of pediatric IBD has been increasing worldwide over the past several years due to unknown but likely multifactorial causes such as changes in environmental risk factors and better diagnostic capabilities.^4^

The current mainstays of IBD treatments include corticosteroids, aminosalicylates, biologics, and small molecule inhibitors. More recently, several investigators have explored the effect of fecal microbial transplant (FMT) in patients with ulcerative colitis with some promising results.^5,6^ These studies have demonstrated how FMT can effectively ameliorate gut dysbiosis and down-regulate inflammation.^7,8^ More specifically, FMT can restore normal microbiota and metabolites including bile acids, short-chain fatty acids, and tryptophan metabolites which have all been implicated in the pathogenesis of IBD.^9^

Bile acids are digestive surfactants that help promote the metabolism of lipids, primarily cholesterol. The primary bile acids, which include cholic acid (CA) and chenodeoxycholic acid (CDCA), are produced in the liver and further conjugated with taurine and glycine prior to storage in the gallbladder.^10^ Following a meal, the gallbladder releases bile acids into the intestinal lumen to help with digestion. 95% of the conjugated BAs are reabsorbed in the distal ileum through enterohepatic circulation, while the remaining 5% remain in the intestines and undergo various microbial transformations including deconjugation, 7α-dehydroxylation, and dehydrogenation.^11^ Deconjugation of these conjugated BAs is considered the “gateway” reaction, opening them to further modification. It is catalyzed by *bile salt hydrolase* (BSH) which is found in multiple gram-positive and gram-negative bacterial genera.

Bile acid profiles following FMT have been studied primarily in subjects presenting with *Clostridium difficile* infection (CDI). Those studies suggest that modification of bile acids following FMT may explain the beneficial effects of FMT.^12^ Additionally, there is emerging data to suggest that the same mechanism may be utilized as a therapy for patients with IBD. For example, in UC patients (without CDI), Paramsothy et al. found that subjects who achieved remission after FMT were more likely to have enrichment of secondary bile acids.^13^

In our previous studies, we demonstrated that bile acid profiles among patients with UC are different compared to those of healthy patients;^14^ healthy patients have a higher level of secondary bile acids when compared to patients with UC.^14^ Now we aim to describe changes in bile acids profiles in children with UC after FMT. In this study, fecal bile acid profiles were measured before FMT and up to 12 months after FMT and compared to healthy children. Furthermore, we identified relationships between fecal bile acid changes, gut bacteria, and genes related to bile acid metabolism.

## Methods

### FMT and sample processing

This study was conducted at Children’s Hospital Los Angeles (CHLA) under IRB # CCI-11-00148 and 16–00050. Witten informed consent was obtained from patients or their legal guardians. Healthy children were asked to complete a questionnaire and provide a single stool sample which was immediately collected and placed in −80°C. Questionnaires included demographics and detailed medical and dietary history. Similar protocol was followed for patients with mild to moderate ulcerative colitis. They provided a baseline sample, prior to any bowel cleanout, then subsequent samples at 4, 12, 24, and 48 weeks following FMT.

Stool samples were processed within 6 hours of collection. For each sample, 50 g of stool content was homogenized with 150 mL of sterile 0.9% normal saline in a commercial blender. The slurry was passed carefully through 2.0 mm, 1.0 mm, 0.5 mm, and 0.25 mm stainless steel laboratory-grade sieves and then centrifuged at 6,000 × *g* for 15 minutes at 4oC using Sorvall SS-34 rotor, and re-suspended in 50 ml of normal saline and immediately frozen at −80°C.

All UC patients were treated with FMT, with a subset receiving autologous FMT (auto-FMT), and the remainder receiving heterologous FMT (hetero-FMT). FMT was performed via colonoscopy after patients underwent appropriate bowel cleanout. During the procedure, the colonoscope was advanced to the cecum and the stool solution was instilled.

### DNA extraction and shotgun metagenomic sequencing

Metagenomic Fecal DNA was extracted using the QIAamp Powerfecal DNA kit (Qiagen, Germantown MD) and following the manufacturer’s instructions. The DNA quantity and quality were checked using Nanodrop. Metagenomic libraries were constructed using the Illumina Nextera XT DNA Library Preparation Kit and Illumina Nextera XT Index v2 Kit A and B following the manufacturer’s protocols and library qualities were assessed on Agilent High Sensitivity DNA Bioanalyzer chips. Libraries were pooled and sequenced on an Illumina NextSeq500 High Output v2 flowcell on an Illumina NextSeq 500 System, producing 2 × 150bp paired-end reads. All samples and control reads were preprocessed and quality-filtered using trim_galore (https://www.bioinformatics.babraham.ac.uk/projects/trim_galore/). Host-derived reads were removed using KneadData (https://bitbucket.org/biobakery/kneaddata).

### Taxonomic and functional metagenomic analysis

The resulting raw reads were checked for quality and trimmed. Taxonomic profiling was done by using CLC genomic workbench version 23 (CLC, Bio-Qiagen, Aarhus, Denmark) pipeline using the UHGG (Unified Human Gastrointestinal Genome) database and filter host reads Homo sapiens (GRCH38) host genome removal. Each mapped read was assigned to a taxon, resulting in a taxonomic abundance table. The functional assignment of the presence and abundance of the enzymes of *bile salt hydrolase* (EC 3.5.1.24), *7α-HSDH, 7β-HSDH* and *Bai* genes *baiA, baiB, baiCD, baiE, baiF, baiG, baiH and baiI* were explored using the same platform CLC Genomic workbench. De novo sequence assembly generated contigs, which were then placed into bins using Bin Pangenomes by Sequence tool. To identify gene and Coding DNA sequences (CDS) on resulting contigs we utilized Annotate CDS with Best DIAMOND HIT tool. Bai enzyme and *Bai* gene databases were downloaded from UniProtKB. Input reads were mapped back to the annotated contigs using the map reads to reference the tool and create an abundance of functional annotation using the Build Functional Profile tool.

### Bile acid extraction

To determine concentrations of the individual primary and secondary bile acids (Table 1), 15-mg aliquots of fecal samples were placed in 2-mL Eppendorf tubes on dry ice and kept frozen. A 10 µL anti-oxidant solution (0.2 mg/ml solution BHT/EDTA in 1:1 MeOH: water) was added. This was enriched with 10 uL of bile acid surrogate (SSTD, 1000 nM) and extracted with 500 uL of cold methanol and stainless-steel grinding balls. The extract was homogenized using GenoGrinder 2 × 30 sec, centrifuged, and the supernatant was transferred to a 1.5-mL Eppendorf tube containing 10 µL 20% glycerol solution in MeOH. A second aliquot of 500 uL of cold methanol was added to the centrifugation pellet. This was homogenized again using GenoGrinder 2 × 30 sec, centrifuged, and the second supernatant was combined with the first one in the 1.5-mL Eppendorf tube. Then, the vials were transferred to Speed-vac and evaporated to dryness. Dry samples were reconstituted for liquid chromatography-mass spectrometry in 100 uL of PHAU/CUDA 100 nM in methanol/ACN 50:50, vortexed for 10 sec, then sonicated for 5 min. The rack of samples was set on wet ice for 15 mins. Then, samples were centrifuged for 3 min at 14,000 rpm followed by transferring the supernatant to a glass insert in an amber HPLC vial. These were stored at −20°C until liquid chromatography-mass spectrometry analysis, which was done on Thermo Vanquish UPLC/AB Sciex Qtrap with targeted MRM method.

**Table 1.** List of the most common bile acid acids and abbreviations used.

Name of bile acid	Class of bile acid	Abbreviation
Cholic acid	Primary	CA
Glycocholic acid	Primary	GCA
Taurocholic acid	Primary	TCA
Chenodeoxycholic Acid	Primary	CDCA
Glycochenodeoxycholic Acid	Primary	GCDCA
Taurochenodeoxycholic acid	Primary	TCDCA
Deoxycholic acid	Secondary	DCA
Glycodeoxycholic acid	Secondary	GDCA
Taurodeoxycholic acid	Secondary	TDCA
Lithocholic acid	Secondary	LCA
Glycolithocholic acid	Secondary	GLCA
Taurolithocholic acid	Secondary	TLCA
Ursodeoxycholic acid	Secondary	UDCA
Glycoursodeoxycholic acid	Secondary	GUDCA
Tauroursodeoxycholic acid	Secondary	TUDCA

### Statistical analysis

The ranges of bile acid and gene concentrations varied widely therefore we used logarithm to transform the data, with 10e-5 added to 0s to avoid infinite values. The ranges of non-zero bacterial genus relative abundances varied from 1.39^*^e10-4 to 0.86. We used logarithm to transform the bile acids data as in most studies.^15^ After log-transformation, the data are more likely to be normally distributed. To prevent infinite values, a pseudo count of 0.65 times the minimum non-zero abundance is added to the zero values before applying the log transformation.^16^ We used Wilcoxon rank sum tests to evaluate statistically significant differences between any two interested groups. Measurements with *p* value less than 0.05 were declared as significant.

## Results

### Participant demographics

Key patient information and demographics can be found in Table 2. In total, we included 28 healthy children, including 10 healthy donors, and 48 pediatric UC patients. All subjects submitted samples at baseline and at 4 weeks post-FMT. During the subsequent time points, not all subjects’ bile acids profiles, Bai genes, and genus data were available due to either missing samples (due to subjects’ voluntary withdrawal from the study) or insufficient DNA quality or quantity for performing Next Generation Sequencing (Figure S1).

**Table 2.** Subject demographics.

	Healthy	UC
Number of patients	28	48
Age (median, in years)	**15**	**17**
Gender (males)	13 (46.4%)	26 (54.2%)
Ethnicity (Non-Hispanic)	29 (100%)	29 (60.4%)
Number of patients with history of CDI*		19(39.6%)
Number of patients with prior hospitalizations		25(52.1%)
Number of patients taking steroids before		3(6.23%)
Number of patients taking 5-aminosalicylates before		20(41.7%)
Number of patients taking immunomodulators before		11(22.9%)
Number of patients on biologic therapy (continued through study)		24(50%)
Number of patients receiving autologous FMT±		12(25%)

*CDI = *Clostridium difficile* infection. ± Two subjects received autologous FMT initially and were switched to heterologous FMT at week 12 and week 4, respectively.

The median age of healthy children was approximately 15 years old, while that of the UC patients was about 17 years old. The two groups had a similar age distribution (Figure S2). Seven out of 10 healthy donors were greater than 21 years old, thus not considered pediatric population. There were differences between the ethnicities for healthy and UC subjects. As shown in Table 2, all of the healthy individuals were non-Hispanics, while 60% of UC patients were non-Hispanics. The age distribution between Hispanic and non-Hispanic patients was similar (Figure S3).

Among UC patients, the average length of diagnosis was about four years and about 40% of them had a history of CDI. No patients had active CDI at time of enrollment, and none of the patients received antibiotics for at least six weeks prior to enrollment. Many of these patients had been on medications prior to FMT including biologic therapy, immunomodulators, 5-aminosalicylates (5-ASA) or steroids, as shown in Table 2. At the time of intervention, 24 patients were on biologic therapy, three patients were on immunomodulators, and no patients were on 5-ASA or steroids. All patients remained on their maintenance medication without changes throughout the entire study, with a single exception of one patient requiring steroids at week 12. None of the patients had a diagnosis of primary sclerosing cholangitis and none were on UCDA therapy. Pediatric Ulcerative Colitis Activity Index (PUCAI) scores were monitored for all patients throughout the study and can be found in Table S1.

### Healthy and UC subjects had significant differences in baseline bile acid profiles

**Figure 1.**
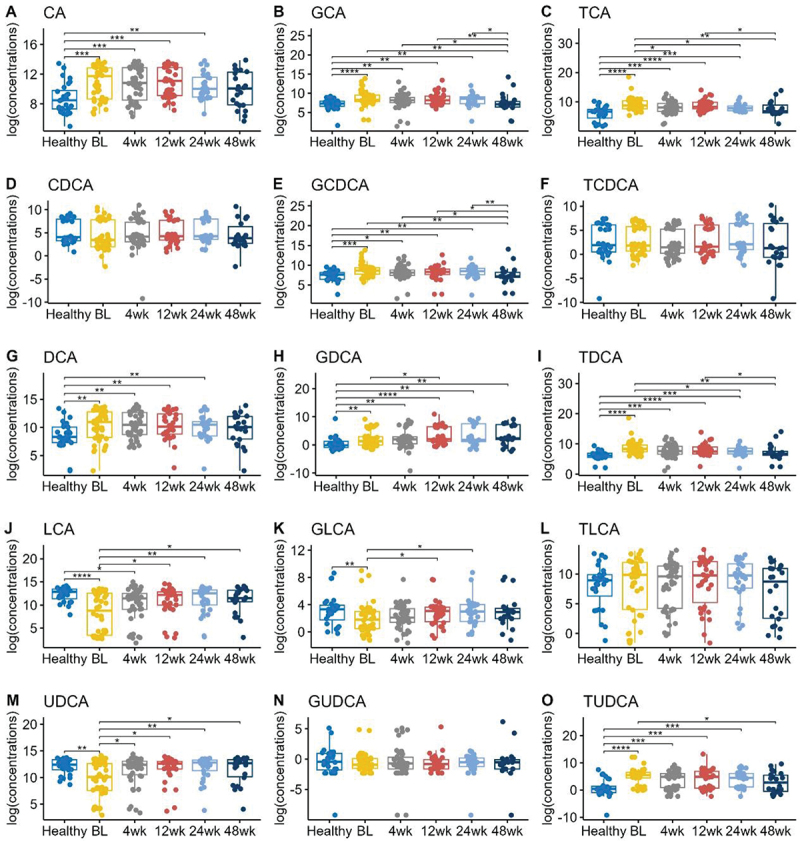
Boxplots of the log transformed concentrations (picograms/milligram feces) of different bile acids in different timepoints. BL = baseline. Pairwise comparisons were performed using Wilcoxon rank sum tests. Only the significant results were annotated in the figure, with *for *p* ≤ 0.05, **for *p* ≤ 0.01, ***for *p* ≤ 0.001, and ****for *p* ≤ 0.0001.

Our previous study demonstrated that children with UC had significantly higher CA, GCA, TCA, GCDCA, DCA, GDCA, TDCA, and TUDCA, and significantly lower CDCA, LCA, GLCA, and UDCA compared to healthy controls.^14^ In this study, we found similar patterns (Figure 1). The primary BAs, CA, GCA, TCA, and GCDCA were increased in UC children at baseline, prior to FMT, compared to healthy controls. Several of the secondary BAs, such as LCA, GLCA and UDCA, were significantly lower in UC children compared to healthy controls.

**Figure 2.**
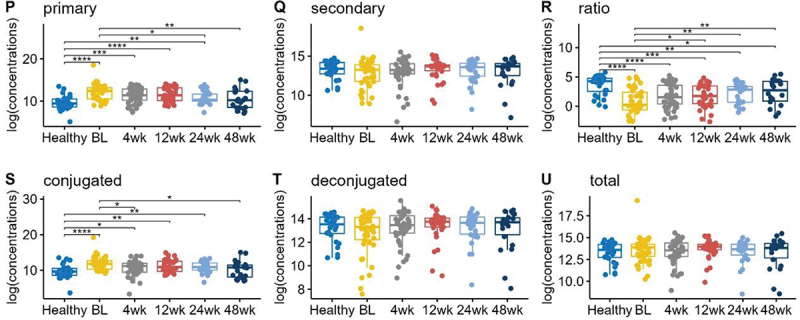
Boxplots of the log transformed concentrations (picograms/milligram feces) of total primary bile acids, total secondary bile acids, the ratio of total secondary to total primary, total conjugated bile acids, total deconjugated bile acids, and total overall bile acids for healthy and UC patients in different time points after FMT. Pairwise comparisons were performed using Wilcoxon rank sum tests. Only the significant results were annotated in the figure, with *for *p* ≤ 0.05, **for *p* ≤ 0.01, ***for *p* ≤ 0.001, and ****for *p* ≤ 0.0001.

Total bile acids were similar in UC children compared to healthy controls, but the ratio of total secondary to total primary BAs was significantly reduced in UC children (Figure 2). This can be attributed to the increase in total primary BAs, as the total secondary BAs did not show any significant difference between UC vs healthy patients. Conjugated BAs were higher in UC patients, while deconjugated BAs were similar. These observations held throughout the 48 week observation period as shown in Figure 2.

### The effects of fecal microbial transplant on bile acid profiles

To evaluate the response of UC patients to FMT, we performed pairwise comparisons using Wilcoxon rank sum tests (Figure 1). At 4 weeks post-FMT, some of the fecal bile acids became more similar to those of healthy patients. For example, UDCA concentrations were substantially decreased in UC patients (median log concentrations 10.11 pg/mg compared to 12.44 pg/mg in healthy patients, p-value = 0.006), then increased back to a level of 12.41 pg/mg by 4 weeks post-FMT (p-value = 0.03). Moreover, these subjects maintained a similar level of median log concentration 12.7 pg/mg at 48 weeks post-FMT (p-value = 0.03)

Over the 48-week period following FMT, several bile acid concentrations exhibited a trend toward normalization to healthy levels. Specifically, primary bile acids such as CA, GCA, TCA, and GCDCA showed a decrease, gradually approaching concentrations seen in healthy individuals. Conversely, secondary bile acids like LCA and GLCA demonstrated an upward trend, gradually increasing toward levels observed in healthy counterparts.

We also compared total bile acid concentrations following FMT in the following categories: total primary bile acids, total secondary bile acids, the ratio of secondary to primary bile acids, total conjugated bile acids, and total deconjugated bile acids (Figure 2). Following FMT, primary BAs in UC patients decreased and became more similar to levels in healthy controls over time. The ratio of secondary to primary BAs increased over 48 weeks post-FMT but did not achieve the same level as healthy controls. Conjugated BAs, which were increased in UC patients at baseline, decreased over 12 months. There were no significant changes among total secondary BAs, deconjugated BAs, and total BAs.

A separate comparison was done between subjects receiving auto-FMT and those receiving hetero-FMT (Figure S4). There were significant differences in CA, TCA, and DCA levels at 4 weeks but they became similar by 12 weeks and beyond.

### Hispanic and non-Hispanic UC patients had similar response to FMT

Since our study included both Hispanic and non-Hispanic patients, we were interested in learning whether ethnicity affects the response to FMT. We compared bile acid profiles at each time point between Hispanic and non-Hispanic UC patients using Wilcoxon rank sum tests.

The concentrations of CA, TCA, GCDCA, DCA, TDCA, and TUDCA in Hispanic subjects were significantly higher than that of non-Hispanics at baseline, but these differences dissipated post-FMT (Figure S5). In a previous study comparing fecal bile acids between Caucasian and Hispanic children with UC, we also demonstrated higher concentrations of CA and other bile acids in the Hispanic group.^17^ The total primary and conjugated BAs were also significantly higher in Hispanic UC patients at baseline, but again this difference did not persist post-FMT.

### CDI history did not influence the response of FMT

We have previously confirmed that UC patients with a history of CDI had significantly lower baseline levels of LCA and UDCA compared to UC patients without a history of CDI^14^ however it remains unknown if CDI infection influences bile acid profiles post FMT. In this current study, we only noted a significant difference in TCA concentrations at 4 weeks post-FMT (Figure S6). Otherwise, there were no differences in median concentrations between UC subjects with and without a history of CDI.

### The genes encoding enzymes critical for bile acid metabolism were decreased in UC patients at baseline and increased following FMT

**Figure 3.**
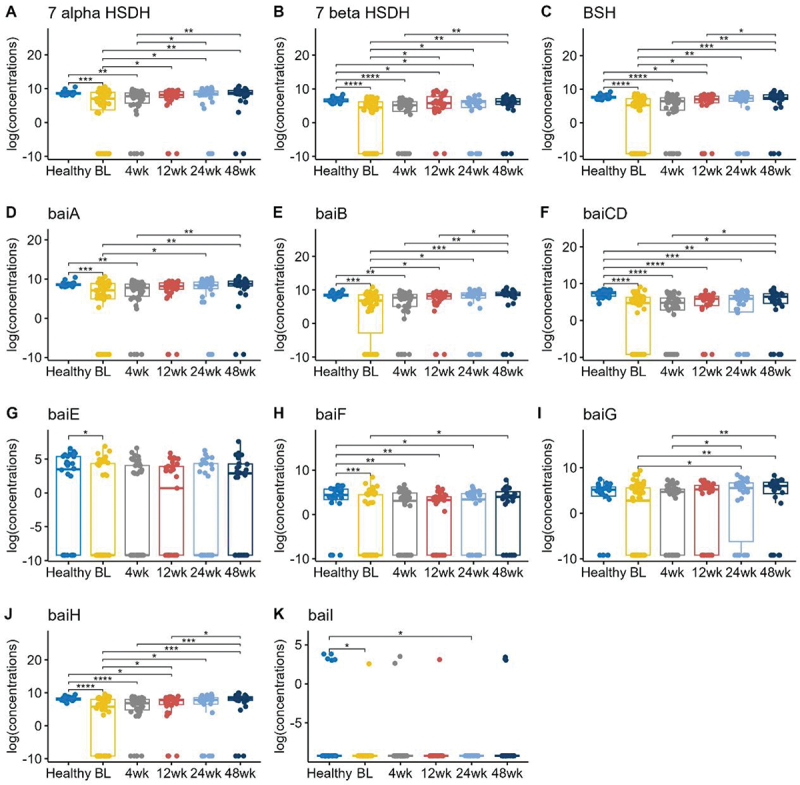
Boxplots of the log transformed relative abundance of different genes responsible for bile acid metabolism. Pairwise comparisons were performed using Wilcoxon rank sum tests. Only the significant results were annotated in the figure, with *for *p* ≤ 0.05, **for *p* ≤ 0.01, ***for *p* ≤ 0.001, and ****for *p* ≤ 0.0001.

We compared the gut microbial genes involved in bile acid metabolism to better understand the changes in fecal BAs in UC patients post-FMT (Figure 3). The genes coding for *bile salt hydrolase (BSH), 7α/β-hydroxysteroid dehydrogenase (HSDH)*, and the *bile-acid-induced (bai) operon* (*baiA, baiB, baiCD, baiE, baiF, baiG, baiH*, and *baiI*) all had decreased abundances in UC patients compared to healthy subjects at baseline, but then increased post-FMT. We reported that UC patients had significantly lower *BSH* (median = 5.17) than healthy subjects (median = 7.58) at baseline (p-value ≤ 0.0001). *BSH* abundances in UC patients (median = 7.42) increased closer to healthy levels (p-value 0.838) at 48 weeks post-FMT. *7α- HSDH, 7β-HSDH*, and most of the *bai* genes showed a similar trend. Of the *bai* operon genes, *baiF* changes were most notable, with *baiF* abundance being undetectable in UC patients with a baseline median of 0, but increased to median of 3.95 at 48 weeks post-FMT, similar to the healthy abundance median of 4.39 (p-value 0.31).

When gene abundances were subdivided into auto-FMT vs hetero-FMT subjects (Figure S7), none of the median concentrations were statistically significantly different at 4 weeks. However, starting at 12 weeks onwards, the auto-FMT group had higher concentrations of *7α-HSDH, 7β-HSDH, baiA, baiB*, and *baiH*. When comparing subgroups of Hispanic vs. non-Hispanic subjects, as well as CDI vs. no CDI history, there were no significant differences (Figures S8-S9).

### Several gut microbial genera exhibited changes associated with bile acid gene changes

**Figure 4.**
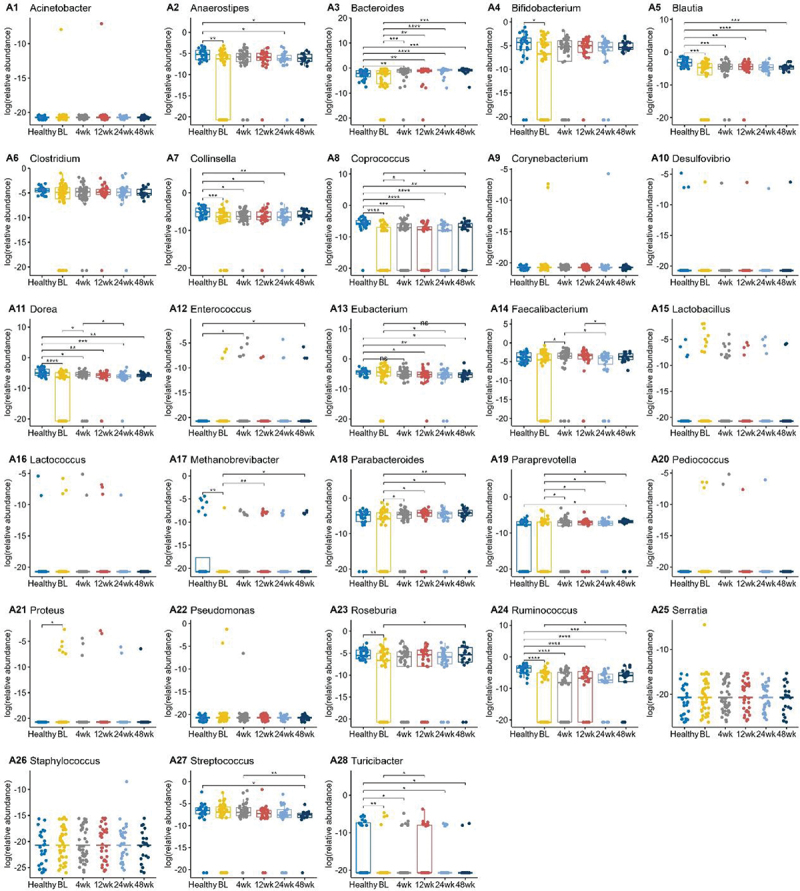
Boxplots of the log transformed relative abundance of different genus at different timepoints. Pairwise comparisons were performed using Wilcoxon rank sum tests. Only the significant results were annotated in the figure, with *for *p* ≤ 0.05, **for *p* ≤ 0.01, ***for *p* ≤ 0.001, and ****for *p* ≤ 0.0001.

Next, we compared levels of bacteria genera involved in bile acid metabolism (Figure 4).^18,19^ The *BSH*-producing genera *Bifidobacterium, Blautia, Dorea, Roseburia*, and *Ruminococcus* were significantly reduced in UC subjects compared to healthy patients. *Bifidobacterium, Dorea, Roseburia*, and *Ruminococcus* levels overall increased following FMT. Other *BSH*-producing genera including *Clostridium, Enterococcus*, and *Lactobacillus* did not show statistically significant differences between UC and healthy patients. *Blautia, Dorea, Roseburia*, and *Ruminococcus* are also involved in 7α dehydroxylation encoded by the *bai* operon. When bacterial genus abundance was compared between the auto-FMT vs hetero-FMT groups, there were only minor differences (Figure S10). The auto-FMT subjects had higher levels of *Bifidobacterium, Collinsella*, and *Parabacteroides* at each of the timepoints, but only the 12-week timepoint was statistically significant. Clostridium levels were higher in the auto-FMT subjects at 4 weeks, but there were no significant differences beyond that. The remaining bacteria genera did not have any statistically significant differences in abundances between the auto- and hetero-FMT groups.

When comparing the Hispanic vs. non-Hispanic groups, there were differences in a few of the bacteria abundances (Figure S11). *Coprococcus* levels were not significantly different at baseline, but at all timepoints post FMT, the Hispanic subjects had lower levels than the non-Hispanic patients. *Ruminococcus* was also found to be lower in Hispanic patients at baseline, 4 weeks, and 12 weeks post FMT.

Significantly lower *Ruminococcus* levels were also seen in subjects with a history of CDI had at baseline, but the difference became negligible by 48 weeks (Figure S12). Levels of *Methanobrevibacter* and *Roseburia* were also different between those with and without a CDI history at some time points (4–12 weeks, and 4 weeks respectively, but again these differences dissipated by 48 weeks post FMT.

## Discussion

Many studies have examined microbiome changes in IBD patients following FMT. Recently, fecal bile acids have emerged as a key metabolite that is altered in patients with IBD, and therefore a potential therapeutic target.^20^ Currently, there is very limited data on fecal bile acid composition and metabolism in IBD patients following FMT. Our study is novel as it is the first pediatric study to compare changes in bile acid composition after FMT. It is also unique given the long-term follow-up duration of 12 months providing insight on duration of bile acid changes.

In this study, we investigated the effects of FMT on bile acid profiles among children with UC. We included patients who received either hetero-FMT or auto-FMT. Auto-FMT is typically used a placebo, but multiple prior studies have considered auto-FMT as a treatment option.^21–23^ Therefore, we included both in our analyses. We observed that certain individual bile acids in the UC group became more similar to the healthy patients post-FMT, with effects lasting up to 12 months. Bile acids and gut microbiota are closely intertwined, so we also delineated corresponding changes in bile genes and gut microorganisms involved in the bile acid metabolism pathway.

Our previous studies along with others have demonstrated that patients with IBD have distinct bile acid profiles compared to healthy controls.^14,24,25^ Our data is consistent with previous data showing that IBD patients have increased total primary BAs and decreased ratio of secondary to primary BAs. The anti-inflammatory properties of secondary BAs are an ongoing area of research, and the decreased ratio of secondary to primary BAs is thought to play a role in intestinal epithelial inflammation.^26^ Therefore, restoring BA composition through FMT could be a potential mechanism by which inflammation could be reduced in IBD.^27^

Although the total secondary BA levels did not change significantly post-FMT, the ratio of secondary to primary BAs did increase in UC patients post-FMT. When examining individual BA concentrations in UC patients post-FMT, the majority became more similar to those seen in healthy controls. This occurred at various time points for each individual BA; some BAs were restored immediately after FMT, while others took several months or even one year to become similar to healthy controls. This is a novel finding since no other study has followed BA changes post-FMT over such an extended time period.

In regard to UC patients with a history of CDI, we previously found that these children had significantly lower LCA and UDCA concentrations compared to UC patients without CDI.^14^ In this study, FMT does not appear to significantly influence bile acid concentrations when comparing subjects with and without CDI. This suggests that the effect of FMT on modifying BAs in children with UC was independent of the status of prior CDI.

We also aimed to compare bile acid profiles at baseline and post-FMT in our subset of Hispanic patients. In general, data on Hispanic children with UC is limited but suggests that they are more likely to develop disease at a younger age, have pan-colonic disease, and undergo colectomy when compared to white patients.^28–30^ There is limited data on microbiome differences in Hispanic patients with UC, and there are currently no studies evaluating bile acid profiles in Hispanic patients post-FMT. The only difference that was identified in Hispanic patients compared to non-Hispanic patients was a baseline elevation in TUDCA. TUDCA has a known chaperone protective effect against endoplasmic reticulum stress and has shown some protective effects in inflammatory gastrointestinal disorders and other degenerative disorders. It has also been shown to increase in patients with ulcerative colitis who have hepatobiliary manifestations^31–37^ By 48 weeks post-FMT, the difference in TUDCA levels in Hispanic vs. non-Hispanic patients dissipated.

**Figure 5.**
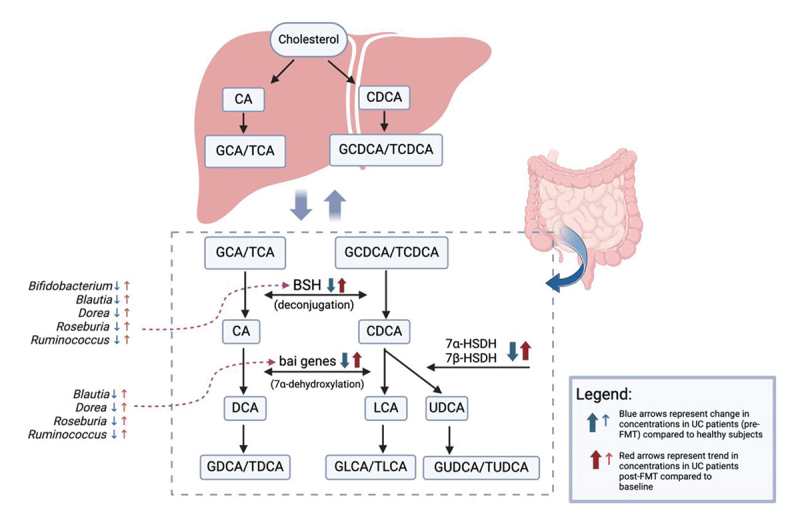
Bile acid metabolism pathways with changes in gene abundances and bacterial genera represented by arrows, as explained in legend. BSH: bile salt hydrolase, 7α-hsdh: 7α- hydroxysteroid dehydrogenase, 7β-hsdh: 7β- hydroxysteroid dehydrogenase, bai: bile acid induced, CA: cholic acid, CDCA: chenodeoxycholic acid, GCA: glycocholic acid, TCA: taurocholic acid, GCDCA: glycochenodexocycholic acid, TCDCA: taurochenodeoxycholic acid, DCA: deoxycholic acid, LCA: lithocholic acid, UDCA: ursodeoxycholic acid, GDCA: glycodeoxycholic acid, TDCA: taurodeoxycholic acid, GLCA: glycolithocholic acid, TLCA: taurolithocholic acid, GUDCA: glycoursodeoxycholic acid, TUDCA: tauroursodeoxycholic acid. Figure created with Biorender.com.

Examining bile acid composition represents just one facet of a broader investigation. In recent years, there has been a surge in research exploring the intricate interplay between bile acid composition, the gut microbiome, and the distinction between healthy and diseased states. Therefore, our next objective was to evaluate how microbial transformations of BAs can change post-FMT. Figure 5 summarizes bile acid metabolism pathways and the changes we identified among gene abundances (*BSH, 7α/7β-HSDH*, and *bai* genes) and their corresponding bacterial genera in UC subjects before and after FMT. Prior to FMT, the increased amounts of primary and conjugated BAs in our UC patients can be explained by the decreased levels of BSH, which in turn corresponded with decreased levels of BSH-containing bacteria including *Bifidobacterium, Blautia, Dorea, Roseburia*, and *Ruminococcus*. Following FMT, we saw these levels approach concentrations closer to those of the healthy patients. These changes persisted over 48 weeks, showing promise for long-term benefits of FMT in reestablishing “healthy” levels of beneficial fecal bile acids and microbes. This is of particular interest since BSH has been directly implicated in intestinal inflammation, and recently shown to inhibit *C. difficile* virulence in murine models.^38^

Another significant reaction in BA metabolism is 7α-dehydroxylation, which is driven by enzymes encoded by the *bai* operon genes (*BaiA* through *BaiH*). These genes are found in *Blautia, Dorea, Roseburia*, and *Ruminococcus*. As previously mentioned, these genera were found to be decreased in UC patients prior to FMT, therefore it is logical that the *bai* genes were also decreased in UC patients. Changes in this pathway also correlated with decreased levels of the downstream bile acid LCA (and its conjugate GLCA) in the UC subjects pre-FMT. Similar to BSH, bai genes have also been explored as a potential target for colitis. In one recent study, mice with mutated *BaiH* exhibited reduced intestinal inflammation.^39^

Although autologous FMT can be considered a placebo, we chose to include these subjects in our main analyses due to the increasing research demonstrating benefits of using auto-FMT as treatment for CDI or IBD. For example, one study by Rossen et al. suggested that autologous FMT could be as effective as heterologous FMT in inducing clinical remission in patients with UC.^23^ Interestingly, the auto-FMT group developed higher concentrations of the genes *7α-HSDH, 7β-HSDH, baiA, baiB*, and *baiH* starting at 12 weeks post FMT.

Our study had a few limitations. We had missing data at some time points as well as loss of follow-up at 48 weeks (up to 50% missing data by 48 weeks, as seen in Figure S1). This is not unusual for a pediatric study that has a long study time. Another limitation was the small size of our subgroups (Hispanic vs. non-Hispanic, CDI history vs no CDI history). Further studies expanding on these subgroups would be helpful in investigating differences between such subgroups.

While our data demonstrated how changes in fecal bile acids relate to changes in enzymatic pathways and associated bacteria, additional studies could expand on the regulatory pathways between microbes and bile acids more directly. Future studies would also be helpful not only to examine a larger cohort, but also to assess how UC disease activity correlates with our observed changes in bile acids following FMT. This could add to existing knowledge and may lead to promising new therapeutic bile acid-related targets for IBD.

## Supplementary Material

Bile Acids FMT_Supplementary Figures_Revised 070124.docx

## Data Availability

The data that support the findings of this study will be made available upon request from the corresponding author, SM.

## References

[1] Bianco AM, Girardelli M, Tommasini A. Genetics of inflammatory bowel disease from multifactorial to monogenic forms. World J Gastroenterol. 2015;21(43):12296–12310. doi:10.3748/wjg.v21.i43.12296. 26604638 PMC4649114

[2] Oliveira SB, Monteiro IM. Diagnosis and management of inflammatory bowel disease in children. BMJ. 2017;357:j2083. doi:10.1136/bmj.j2083. 28566467 PMC6888256

[3] Burgess CJ, Henderson P, Jones G, Lees CW, Wilson DC, Lothian IBD Registry Group. Paediatric patients (less than age of 17 years) account for less than 1.5% of all prevalent inflammatory bowel disease cases. J Pediatr Gastroenterol Nutr. 2020;71(4):521–523. doi:10.1097/MPG.0000000000002842. 32639452

[4] Kuenzig ME, Fung SG, Marderfeld L, Mak JWY, Kaplan GG, Ng SC, Wilson DC, Cameron F, Henderson P, Kotze PG, et al. Twenty-first century trends in the global epidemiology of pediatric-onset inflammatory bowel disease: systematic review. Gastroenterology. 2022;162(4):1147–1159.e4. doi:10.1053/j.gastro.2021.12.282. 34995526

[5] Cheng F, Huang Z, Li Z, Wei W. Efficacy and safety of fecal microbiota transplant for recurrent Clostridium difficile infection in inflammatory bowel disease: a systematic review and meta-analysis. Rev Esp Enferm Dig. 2022;114(9):543–549. doi:10.17235/reed.2022.8814/2022. 35510325

[6] Vuyyuru SK, Kedia S, Kalaivani M, Sahu P, Kante B, Kumar P, Ranjan MK, Makharia G, Ananthakrishnan A, Ahuja V. Efficacy and safety of fecal transplantation versus targeted therapies in ulcerative colitis: network meta-analysis. Future Microbiol. 2021;16(15):1215–1227. doi:10.2217/fmb-2020-0242. 34590904

[7] Stallmach A, Grunert P, Pieper D, Steube A. Ulcerative colitis: does the modulation of gut microbiota induce long-lasting remission? Z Gastroenterol. 2019;57(7):834–842. doi:10.1055/a-0874-6603. 30986885

[8] Zhang W, Jin Z, Yang Z, Zhang J-Y, Ma X-H, Guan J, Sun B-L, Chen X. Fecal microbiota transplantation ameliorates active ulcerative colitis by Downregulating Pro-inflammatory Cytokines in mucosa and serum. Front Microbiol. 2022;13:818111. doi:10.3389/fmicb.2022.818111. 35444617 PMC9014222

[9] Lavelle A, Sokol H. Gut microbiota-derived metabolites as key actors in inflammatory bowel disease. Nat Rev Gastroenterol Hepatol. 2020;17(4):223–237. doi:10.1038/s41575-019-0258-z. 32076145

[10] Staels B, Fonseca VA. Bile acids and metabolic regulation: mechanisms and clinical responses to bile acid sequestration. Diabetes Care. 2009;32(Suppl 2):237. doi:10.2337/dc09-S355. PMC2811459 19875558

[11] Staley C, Weingarden AR, Khoruts A, Sadowsky MJ. Interaction of gut microbiota with bile acid metabolism and its influence on disease states. Appl Microbiol Biotechnol. 2017;101(1):47–64. doi:10.1007/s00253-016-8006-6. 27888332 PMC5203956

[12] Mullish BH, McDonald JAK, Pechlivanis A, Allegretti JR, Kao D, Barker GF, Kapila D, Petrof EO, Joyce SA, Gahan CGM, et al. Microbial bile salt hydrolases mediate the efficacy of faecal microbiota transplant in the treatment of recurrent Clostridioides difficile infection. Gut. 2019;68(10):1791–1800. doi:10.1136/gutjnl-2018-317842. 30816855 PMC6839797

[13] Paramsothy S, Nielsen S, Kamm MA, Deshpande NP, Faith JJ, Clemente JC, Paramsothy R, Walsh AJ, van den Bogaerde J, Samuel D, et al. Specific bacteria and metabolites associated with response to fecal microbiota transplantation in patients with ulcerative colitis. Gastroenterology. 2019;156(5):1440–1454.e2. doi: 10.1053/j.gastro.2018.12.001. 30529583

[14] Rotondo-Trivette S, Wang B, Gayer C, Parsana R, Luan Y, Sun F, Michail S. Decreased secondary faecal bile acids in children with ulcerative colitis and clostridioides difficile infection. Aliment Pharmacol Ther. 2021;54(6):792–804. doi:10.1111/apt.16496. 34218431 PMC8384671

[15] van Best N, Rolle-Kampczyk U, Schaap FG, van Best N, Basic M, Olde Damink SWM, Bleich A, Savelkoul PHM, von Bergen M, Penders J, et al. Bile acids drive the newborn’s gut microbiota maturation. Nat Commun. 2020;11(1):3692–3698. doi:10.1038/s41467-020-17183-8. 32703946 PMC7378201

[16] Martín-Fernández JA, Barceló-Vidal C, Pawlowsky-Glahn V. Dealing with zeros and missing values in compositional data sets using nonparametric imputation. Math Geol. 2003;35(3):253–278. doi:10.1023/A:1023866030544.

[17] Aboud Syriani L, Parsana R, Durazo-Arvizu RA, Michail S. Differences in gut microbiota and fecal bile acids between Caucasian and Hispanic children and young adults with ulcerative colitis. Physiol Rep. 2023;11(12):e15752. doi:10.14814/phy2.15752. 37344396 PMC10284820

[18] Guzior DV, Quinn RA. Review: microbial transformations of human bile acids. Microbiome. 2021;9(1):140–141. doi:10.1186/s40168-021-01101-1. 34127070 PMC8204491

[19] Song Z, Feng S, Zhou X, Song Z, Li J, Li P. Taxonomic identification of bile salt hydrolase-encoding lactobacilli: modulation of the enterohepatic bile acid profile. iMeta. 2023;2(3):e128. doi:10.1002/imt2.128. 38867937 PMC10989828

[20] Yang M, Gu Y, Li L, Liu T, Song X, Sun Y, Cao X, Wang B, Jiang K, Cao H, et al. Bile acid–gut microbiota axis in inflammatory bowel disease: from bench to bedside. Nutrients. 2021;13(9):3143. doi: 10.3390/nu13093143. 34579027 PMC8467364

[21] Basson AR, Zhou Y, Seo B, Rodriguez-Palacios A, Cominelli F. Autologous fecal microbiota transplantation for the treatment of inflammatory bowel disease. Transl Res. 2020;226:1–11. doi:10.1016/j.trsl.2020.05.008. 32585148 PMC7308243

[22] Kelly CR, Khoruts A, Staley C, Sadowsky MJ, Abd M, Alani M, Bakow B, Curran P, McKenney J, Tisch A, et al. Effect of fecal microbiota transplantation on recurrence in multiply recurrent clostridium difficile infection: a randomized trial. Ann Intern Med. 2016;165(9):609–616. doi:10.7326/M16-0271. 27547925 PMC5909820

[23] Rossen NG, Fuentes S, van der Spek MJ, Tijssen JG, Hartman JHA, Duflou A, Löwenberg M, van den Brink GR, Mathus-Vliegen EMH, de Vos WM, et al. Findings from a randomized controlled trial of fecal transplantation for patients with ulcerative colitis. Gastroenterology. 2015;149(1):110–118.e4. doi:10.1053/j.gastro.2015.03.045. 25836986

[24] Duboc H, Rajca S, Rainteau D, Benarous D, Maubert M-A, Quervain E, Thomas G, Barbu V, Humbert L, Despras G, et al. Connecting dysbiosis, bile-acid dysmetabolism and gut inflammation in inflammatory bowel diseases. Gut. 2013;62(4):531–539. doi:10.1136/gutjnl-2012-302578. 22993202

[25] Franzosa EA, Sirota-Madi A, Avila-Pacheco J, Fornelos N, Haiser HJ, Reinker S, Vatanen T, Hall AB, Mallick H, McIver LJ, et al. Gut microbiome structure and metabolic activity in inflammatory bowel disease. Nat Microbiol. 2019;4(2):293–305. doi:10.1038/s41564-018-0306-4. 30531976 PMC6342642

[26] Sinha SR, Haileselassie Y, Nguyen LP, Tropini C, Wang M, Becker LS, Sim D, Jarr K, Spear ET, Singh G, et al. Dysbiosis-induced secondary bile acid deficiency promotes intestinal inflammation. Cell Host & Microbe. 2020;27(4):659–670.e5. doi:10.1016/j.chom.2020.01.021. 32101703 PMC8172352

[27] Chunhua Z, Ying W, Cun L, Zhiyong X, Lei D, Tang X. Amelioration of colitis by a gut bacterial consortium producing anti-inflammatory secondary bile acids. Microbiol Spectr. 2023;11(2):3330. doi:10.1128/spectrum.03330-22. PMC10101101 36943054

[28] Nguyen GC, Torres EA, Regueiro M, Bromfield G, Bitton A, Stempak J, Dassopoulos T, Schumm P, Gregory FJ, Griffiths AM, et al. Inflammatory bowel disease characteristics among African americans, hispanics, and non-Hispanic whites: characterization of a large North American cohort. Am J Gastroenterol. 2006;101(5):1012–1023. doi:10.1111/j.1572-0241.2006.00504.x. 16696785

[29] Afzali A, Cross RK. Racial and ethnic minorities with inflammatory bowel disease in the United States: a systematic review of disease characteristics and differences. Inflamm Bowel Dis. 2016;22(8):2023–2040. doi:10.1097/MIB.0000000000000835. 27379446

[30] Hattar LN, Abraham BP, Malaty HM, Smith EO, Ferry GD. Inflammatory bowel disease characteristics in Hispanic children in Texas. Inflamm Bowel Dis. 2012;18(3):546–554. doi:10.1002/ibd.21698. 21456045

[31] Wong W, Chan BD, Sham T, Lee MML, Chan C-O, Chau C-T, Mok DKW, Kwan Y-W, Tai WCS. Lactobacillus casei strain shirota ameliorates dextran sulfate sodium-induced colitis in mice by increasing taurine-conjugated bile acids and inhibiting nf-κB signaling via stabilization of IκBα. Front Nutr. 2022;9:816836. doi:10.3389/fnut.2022.816836. 35529468 PMC9069136

[32] Huang F, Pariante CM, Borsini A. From dried bear bile to molecular investigation: a systematic review of the effect of bile acids on cell apoptosis, oxidative stress and inflammation in the brain, across pre-clinical models of neurological, neurodegenerative and neuropsychiatric disorders. Brain Behav Immun. 2022;99:132–146. doi:10.1016/j.bbi.2021.09.021. 34601012

[33] Kim SH, Kim JW, Koh S, Kim SG, Bae JM, Kim JH, Park JH, Chang MS, Choi KD, Kang HW, et al. Tauroursodeoxycholic acid inhibits nuclear factor kappa B signaling in gastric epithelial cells and ameliorates gastric mucosal damage in mice. Korean J Gastroenterol. 2022;79(4):161–169. doi:10.4166/kjg.2022.003. 35473774 PMC12286233

[34] Kim YH, Kim JH, Kim BG, Lee KL, Kim JW, Koh S. Tauroursodeoxycholic acid attenuates colitis-associated colon cancer by inhibiting nuclear factor kappaB signaling. J Gastroenterol Hepatol. 2019;34(3):544–551. doi:10.1111/jgh.14526. 30378164

[35] Wang W, Zhao J, Gui W, Sun D, Dai H, Xiao L, Chu H, Du F, Zhu Q, Schnabl B, et al. Tauroursodeoxycholic acid inhibits intestinal inflammation and barrier disruption in mice with non-alcoholic fatty liver disease. Br J Pharmacol. 2018;175(3):469–484. doi:10.1111/bph.14095. 29139555 PMC5773980

[36] den Bossche L V, Borsboom D, Devriese S, Van Welden S, Holvoet T, Devisscher L, Hindryckx P, De Vos M, Laukens D. Tauroursodeoxycholic acid protects bile acid homeostasis under inflammatory conditions and dampens Crohn’s disease-like ileitis. Lab Invest. 2017;97(5):519–529. doi:10.1038/labinvest.2017.6. 28165466

[37] Gnewuch C, Liebisch G, Langmann T, Dieplinger B, Mueller T, Haltmayer M, Dieplinger H, Zahn A, Stremmel W, Rogler G, et al. Serum bile acid profiling reflects enterohepatic detoxification state and intestinal barrier function in inflammatory bowel disease. World J Gastroenterol. 2009;15(25):3134–3141. doi:10.3748/wjg.15.3134. 19575493 PMC2705736

[38] Foley MH, Walker ME, Stewart AK, O’Flaherty S, Gentry EC, Patel S, Beaty VV, Allen G, Pan M, Simpson JB, et al. Bile salt hydrolases shape the bile acid landscape and restrict clostridioides difficile growth in the murine gut. Nat Microbiol. 2023;8(4):611–628. doi:10.1038/s41564-023-01337-7. 36914755 PMC10066039

[39] Jin W, Li T, Huo D, Qu S, Li XV, Arifuzzaman M, Lima SF, Shi H-Q, Wang A, Putzel GG, et al. Genetic manipulation of gut microbes enables single-gene interrogation in a complex microbiome. Cell. 2022;185(3):547–562.e22. doi:10.1016/j.cell.2021.12.035. 35051369 PMC8919858

