# Supplementary material for: Fecal bile acid profiles before and after fecal microbial transplant in pediatric onset ulcerative colitis
Source: Gut Microbes Rep. 2024 Sep 27;1(1):2393219. doi: 10.1080/29933935.2024.2393219 (PMC12306628; doi:10.1080/29933935.2024.2393219)
Supplement: Bile Acids FMT_Supplementary Figures_Revised 070124.docx [file KGMR_A_2393219_SM4576.docx]

Supplemental Material


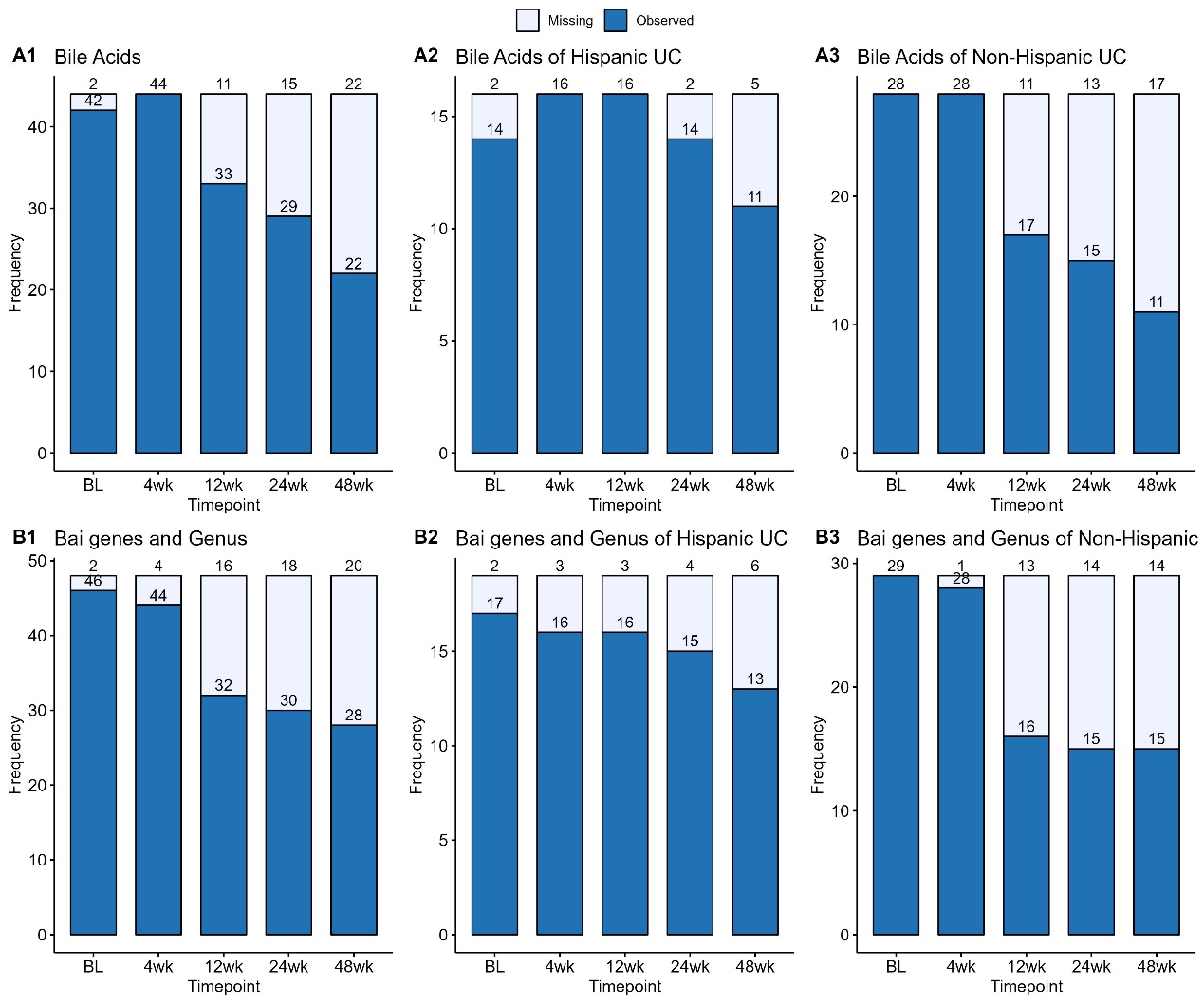


**Figure S1:** Frequency of missing and observed samples at different timepoints for various data types employed in the analysis. For each data type, a representative quantitative measure was presented and other variables within the same data type exhibit identical data patterns. (A1) Missing and observed samples for bile acid data, represented by the CA; (A2) Missing and observed samples for bile acid data of Hispanic UC, represented by the CA; (A3) Missing and observed samples for bile acid data of Non-Hispanic UC, represented by the CA; (B1) Missing and observed samples for bai genes and genus data, represented by the *baiA*; (B2) Missing and observed samples for bai genes and genus data of Hispanic UC, represented by the *baiA*; (B3) Missing and observed samples for bai genes and genus data of Non-Hispanic UC, represented by the *baiA*.


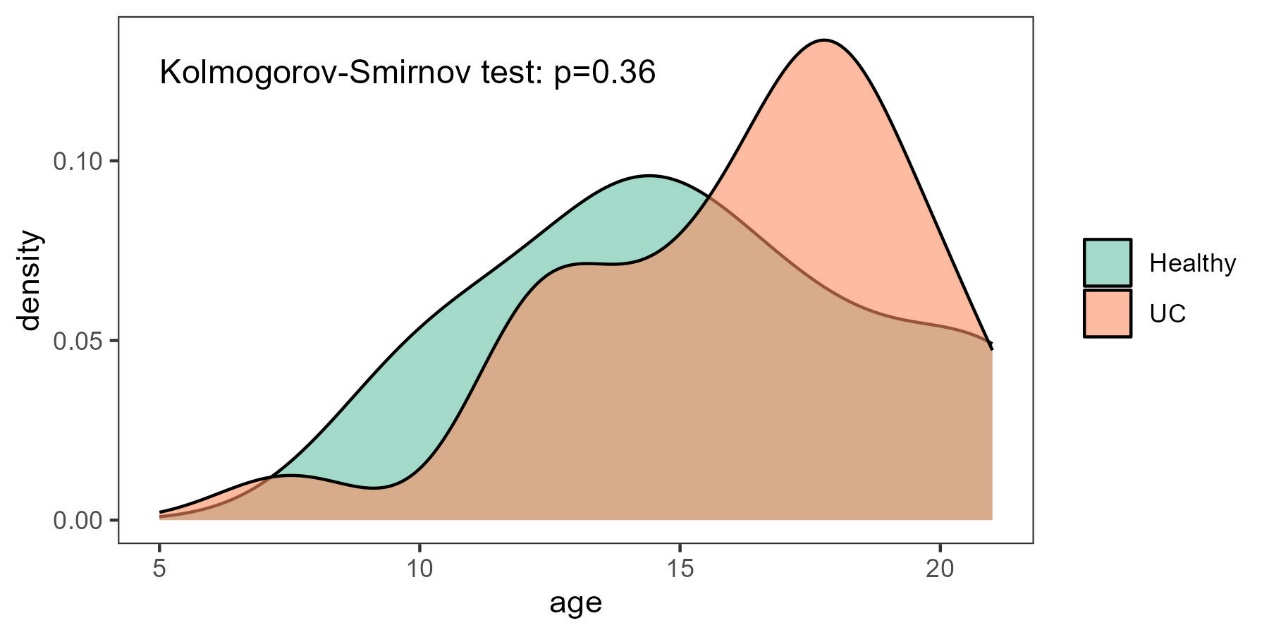


Figure S2: Age distribution for Healthy and UC subjects. P value from Kolmogorov-Smimov test is greater than 0.05, indicating subjects in two groups had similar distribution.


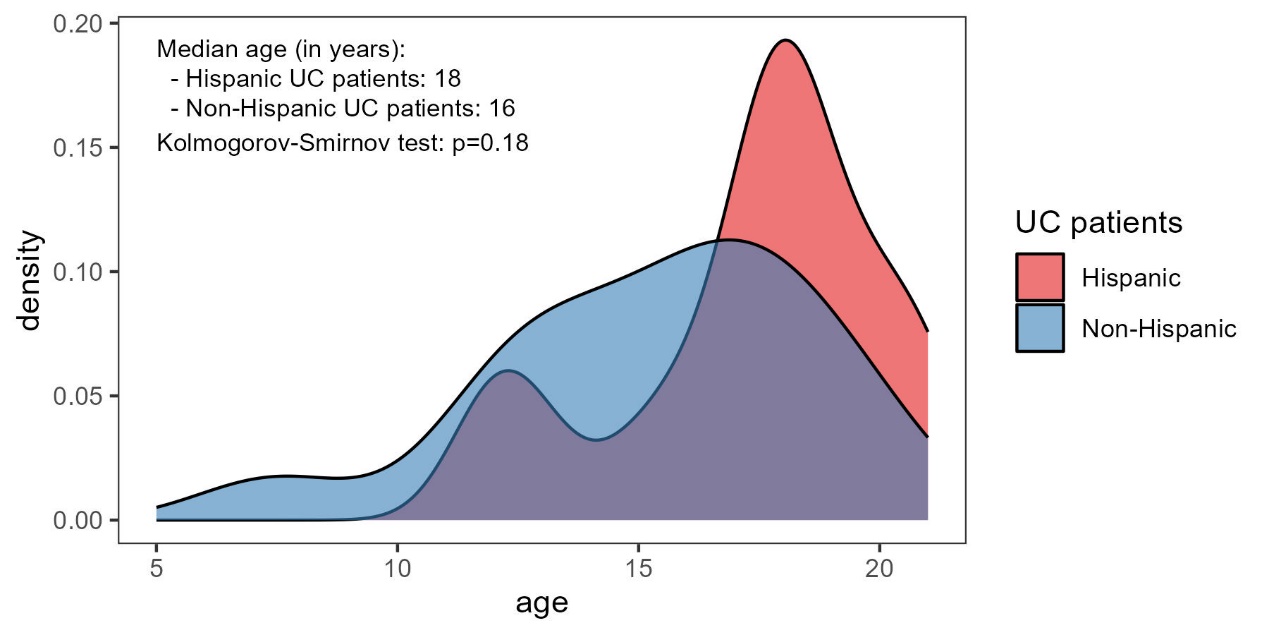


Figure S3: Age distribution comparison between Hispanic UC and Non-Hispanic UC patients. Despite a median age of 18 for Hispanic UC patients and 16 for Non-Hispanic UC patients, the p-value from the Kolmogorov-Smirnov test suggests that the age distribution of patients in these two groups is similar.

| Timepoint | PUCAI | PUCAI for auto-FMT  (mean$\pm$sd) | PUCAI for hetero-FMT  (mean$\pm$sd) | p-value between auto-FMT and hetero-FMT groups |
| --- | --- | --- | --- | --- |
| BL | 39$\pm$16.3 | 41.8$\pm$17.2 | 38.9$\pm$15.8 | 0.498 |
| 4wk | 20.2$\pm$17.3 | 16.8$\pm$15.5 | 21.3$\pm$17.9 | 0.568 |
| 12wk | 15.7$\pm$15.3 | 8.9$\pm$13.2 | 18.2$\pm$15.5 | 0.046 |
| 24wk | 13.5$\pm$14.9 | 8.1$\pm$7.5 | 15.5$\pm$16.5 | 0.353 |
| 48wk | 10.8$\pm$13.1 | 3.8$\pm$5.2 | 13.4$\pm$14.2 | 0.063 |

Table S1: PUCAI scores for all UC patients at different timepoints, as well as comparing those who received auto-FMT vs hetero-FMT. The differences between two groups were tested using Wilcoxon test.


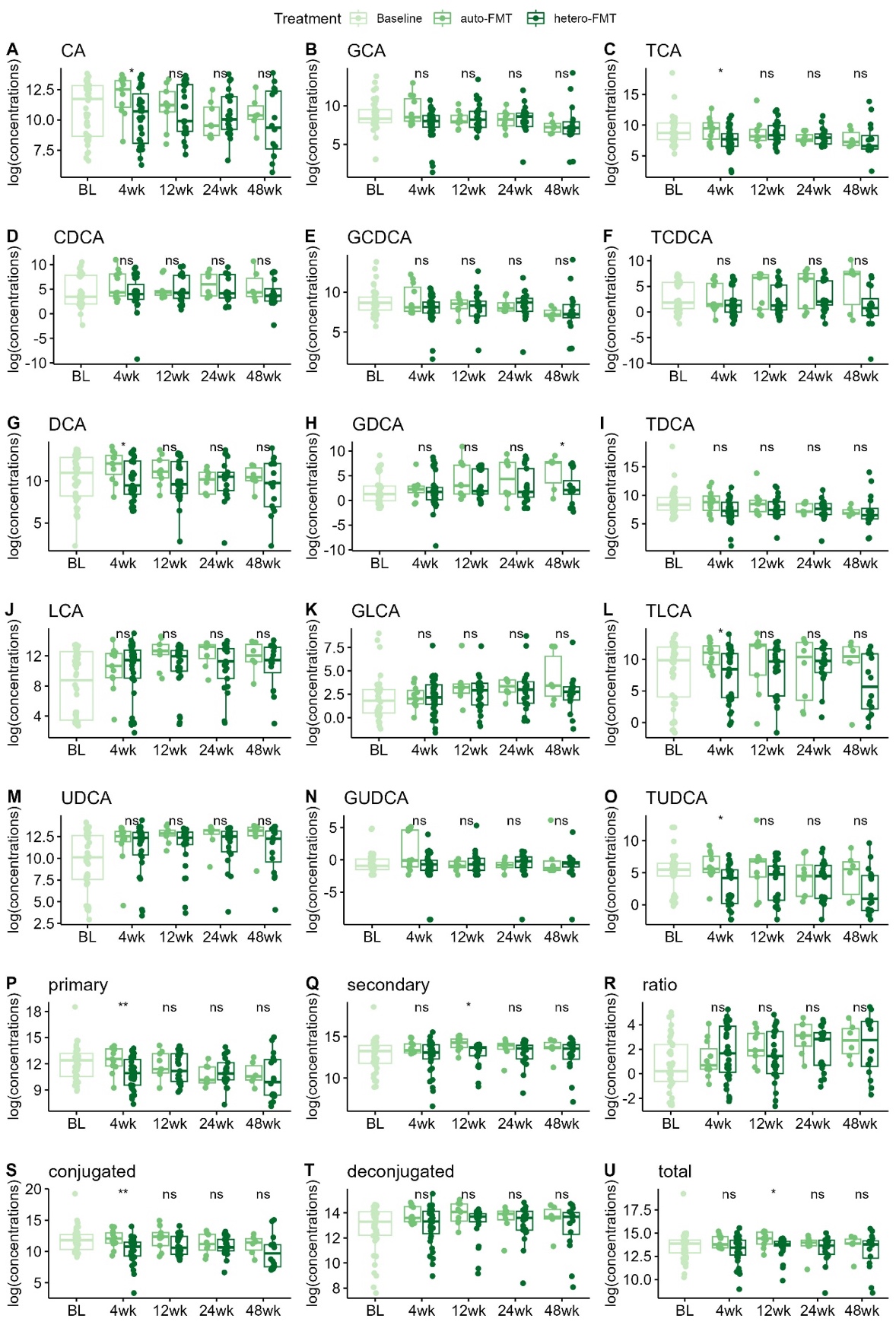


**Figure S4:** Boxplots of the log transformed concentrations (picograms/milligram feces) of different bile acids comparing patients receiving auto-FMT and hetero-FMT. Pairwise comparisons were performed using Wilcoxon rank sum tests, with ns (not significant) for p>0.05, * for p <= 0.05, ** for p <= 0.01, *** for p <= 0.001, and **** for p <= 0.0001.


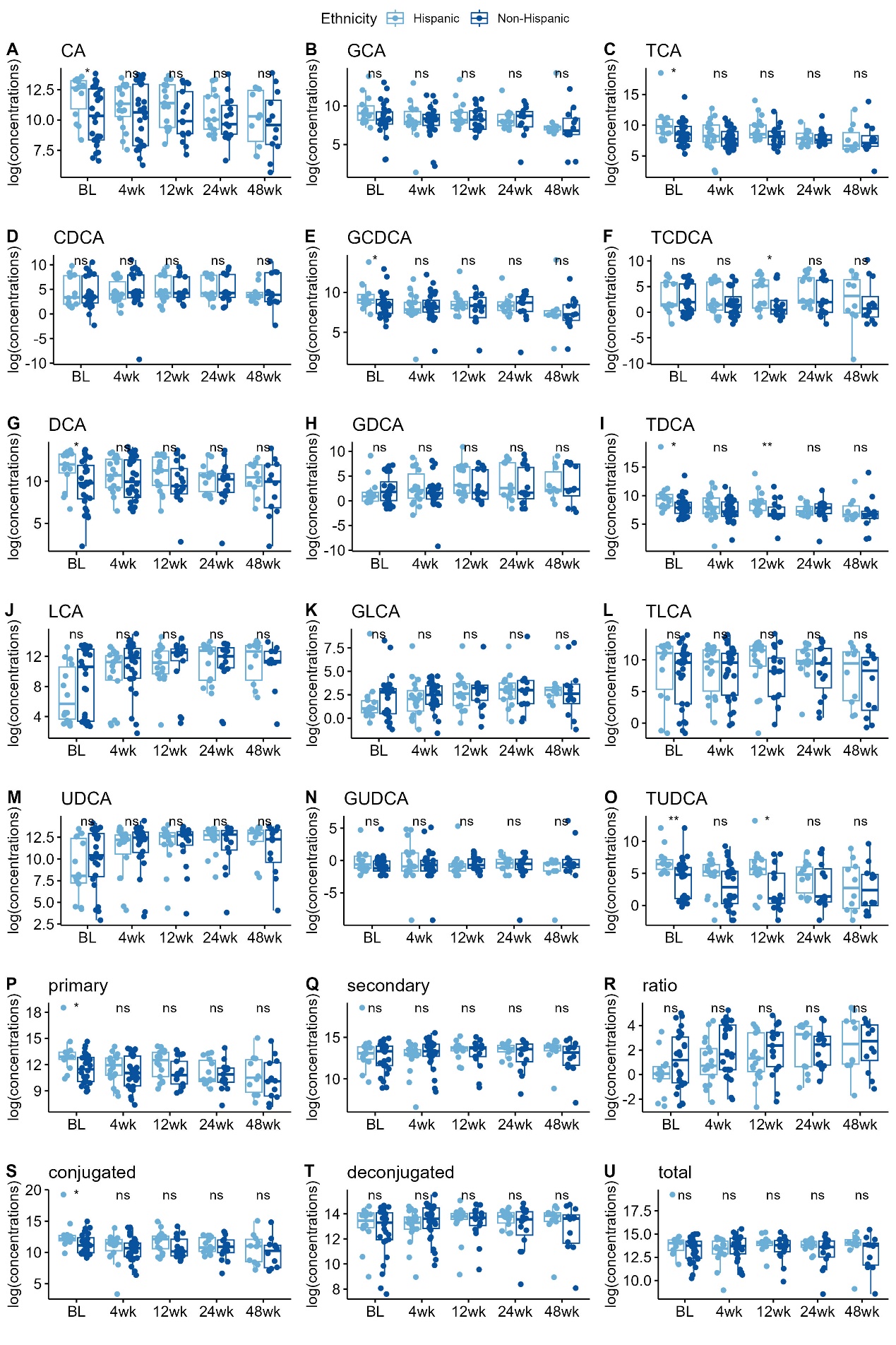


**Figure S5:** Boxplots of the log transformed concentrations (picograms/milligram feces) of bile acids in Hispanic vs non-Hispanic UC patients. Pairwise comparisons were performed using Wilcoxon rank sum tests, with ns (not significant) for p>0.05, * for p <= 0.05, ** for p <= 0.01, *** for p <= 0.001, and **** for p <= 0.0001.


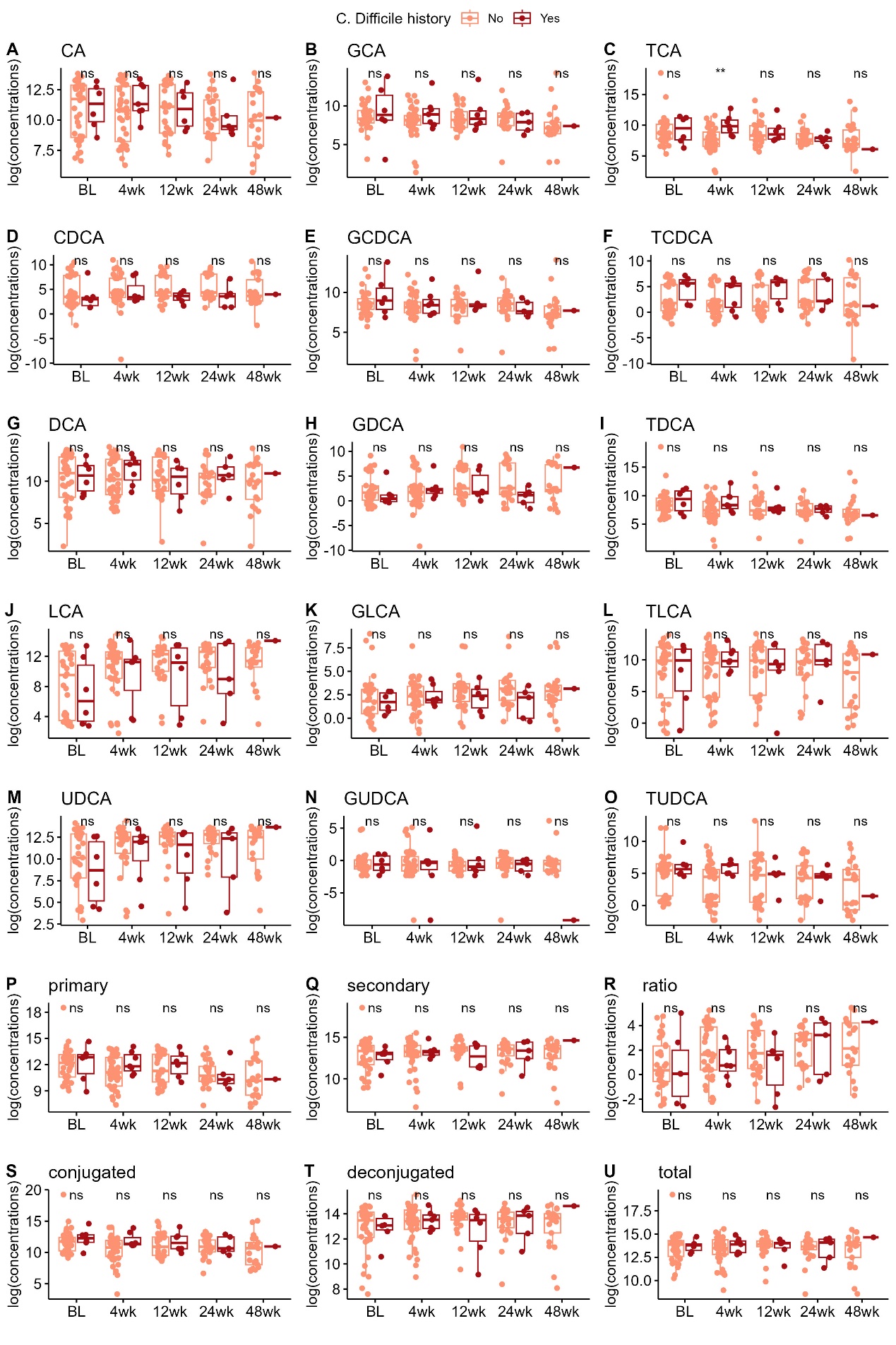


**Figure S6:** Boxplots of the log transformed concentrations (picograms/milligram feces) of bile acids distinguished by *C. difficile* history. Pairwise comparisons were performed using Wilcoxon rank sum tests, with ns (not significant) for p>0.05, * for p <= 0.05, ** for p <= 0.01, *** for p <= 0.001, and **** for p <= 0.0001.


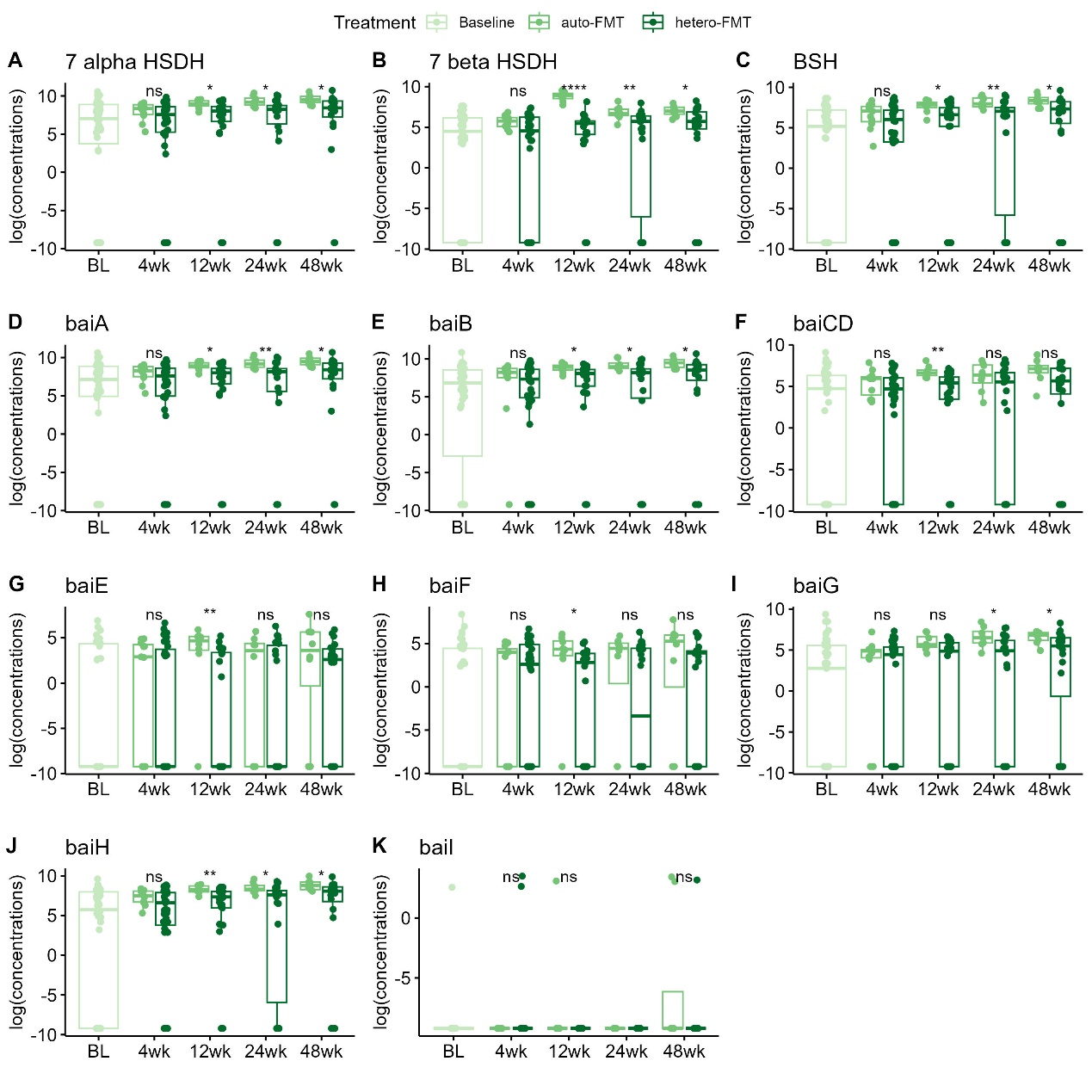


**Figure S7:** Boxplots of the log transformed relative abundance of different genes based on auto-FMT vs hetero-FMT. Pairwise comparisons were performed using Wilcoxon rank sum tests, with ns (not significant) for p>0.05, * for p <= 0.05, ** for p <= 0.01, *** for p <= 0.001, and **** for p <= 0.0001.


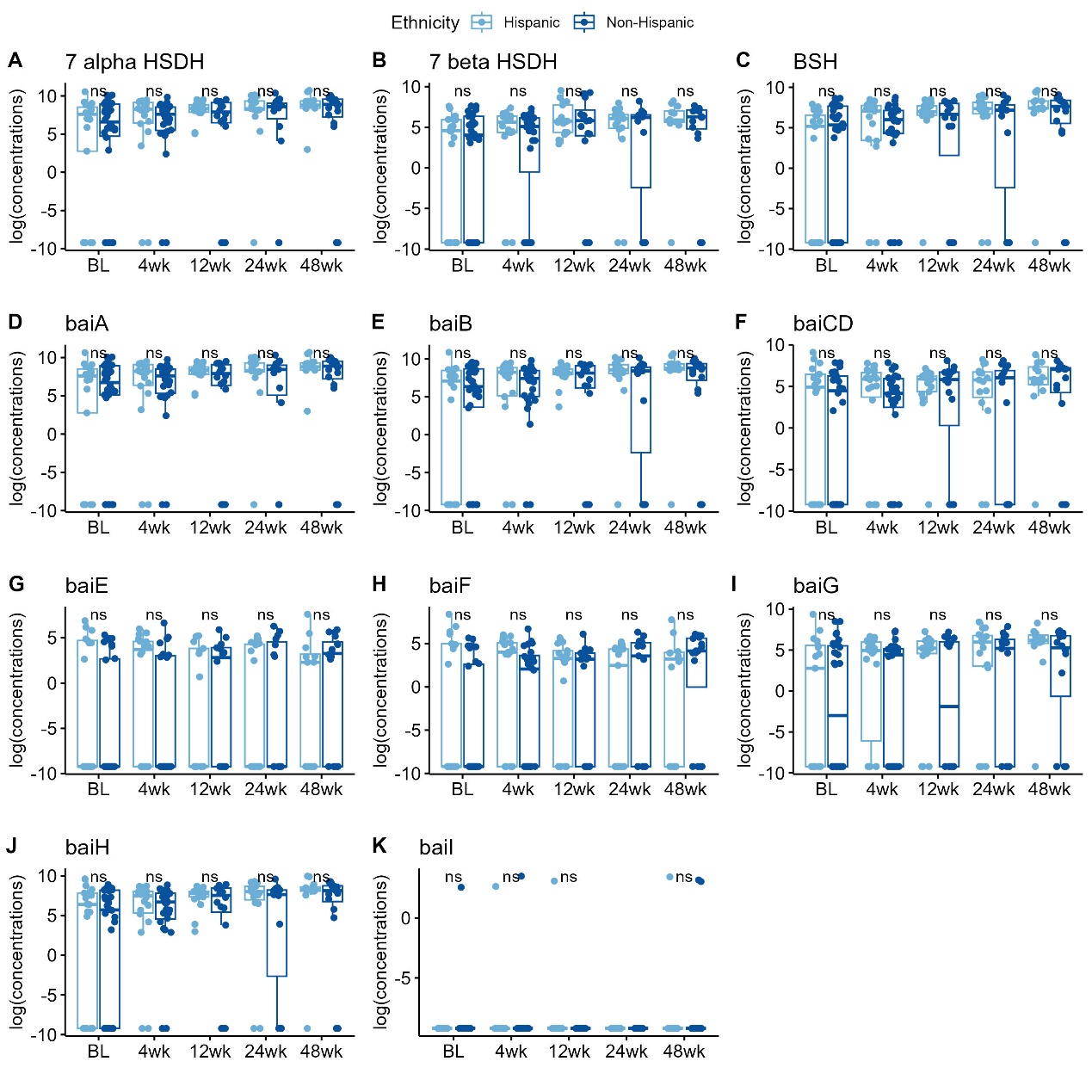


**Figure S8:** Boxplots of the log transformed concentrations (picograms/milligram feces) of different bai genes in different ethnicities. Pairwise comparisons were performed using Wilcoxon rank sum tests, with ns (not significant) for p>0.05, * for p <= 0.05, ** for p <= 0.01, *** for p <= 0.001, and **** for p <= 0.0001.


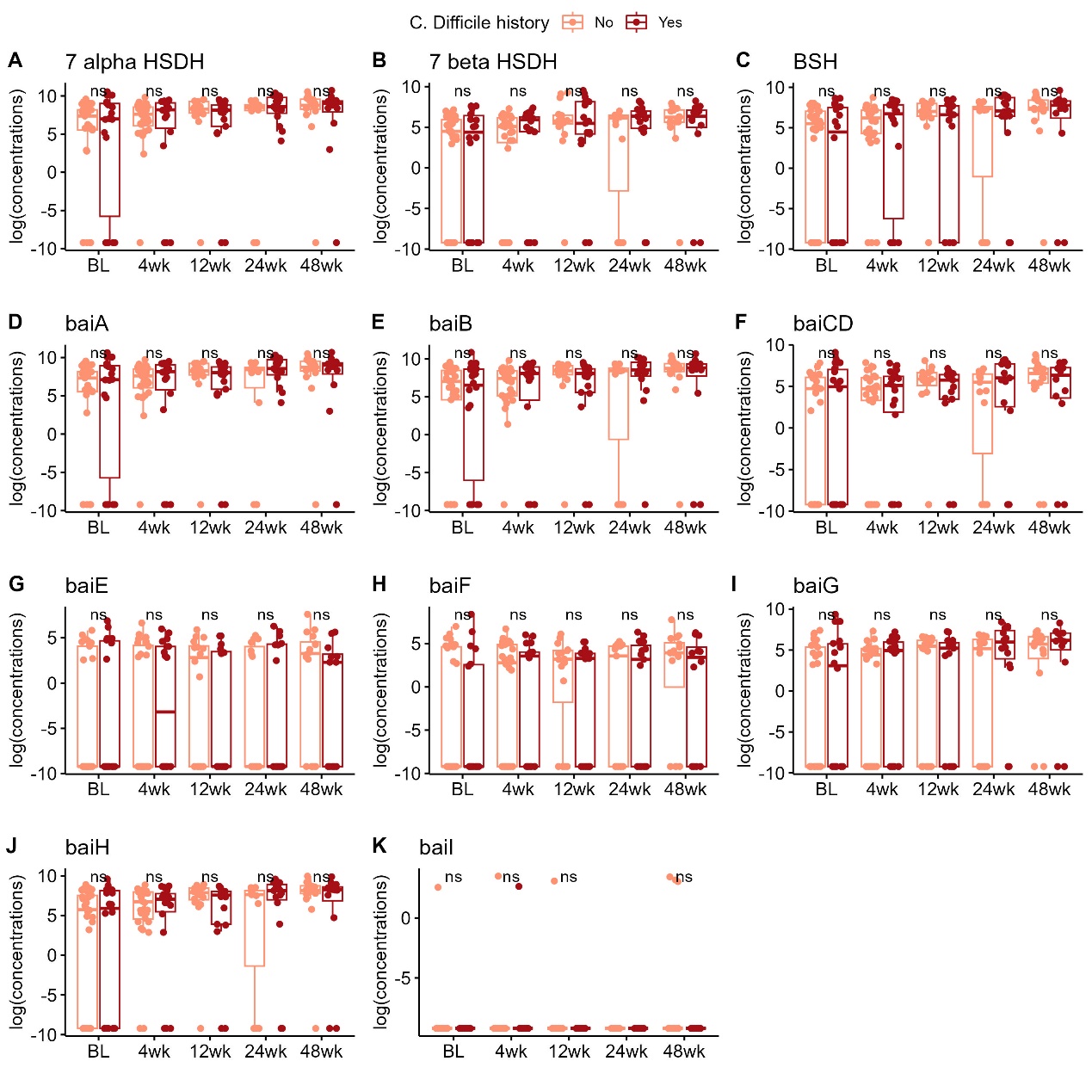


**Figure S9:** Boxplots of the log transformed concentrations (picograms/milligram feces) of different bai genes based on history of C. difficile infection. Pairwise comparisons were performed using Wilcoxon rank sum tests, with ns (not significant) for p>0.05, * for p <= 0.05, ** for p <= 0.01, *** for p <= 0.001, and **** for p <= 0.0001.


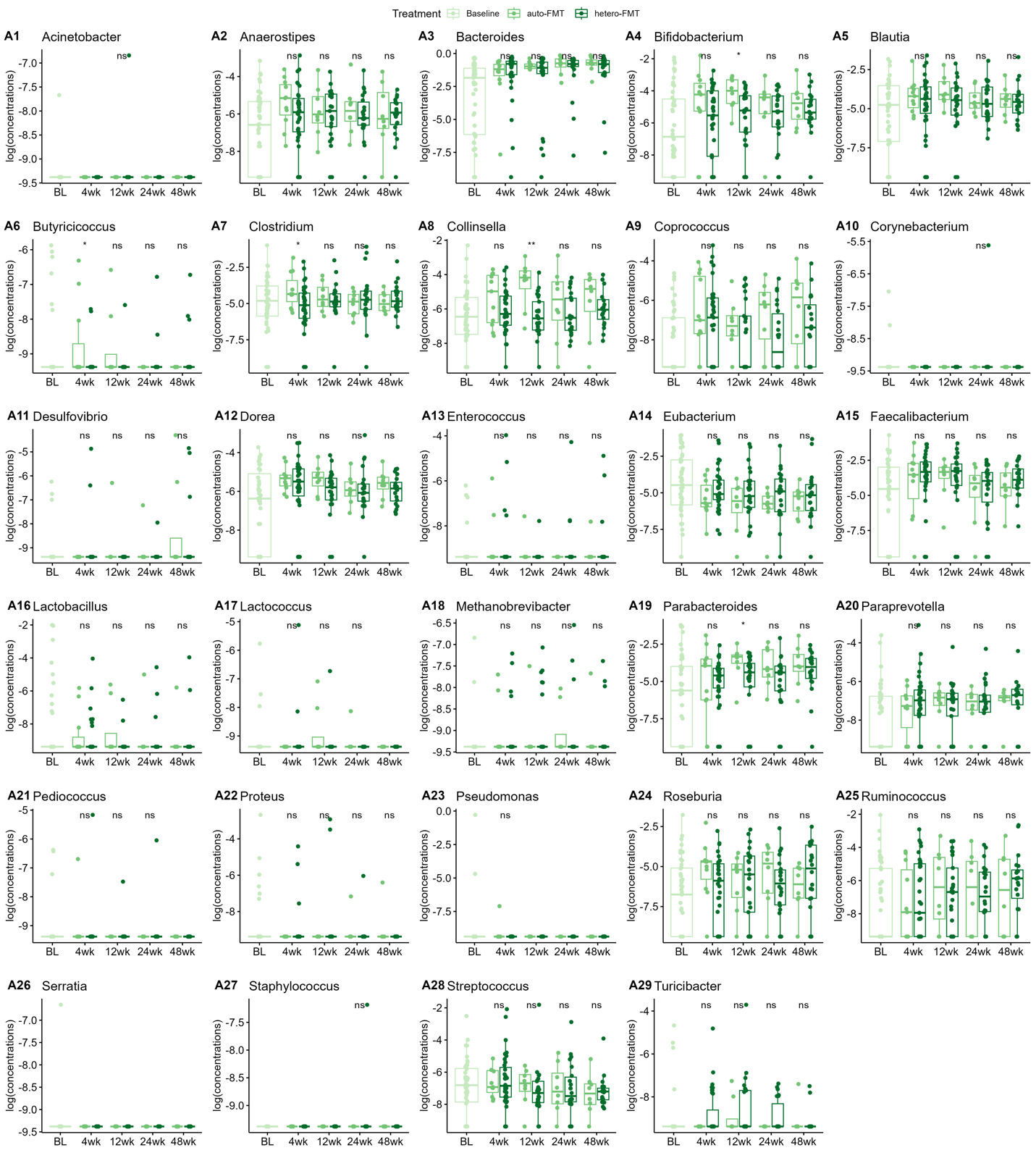


**Figure S10**: Boxplots of the log transformed relative abundance of different genus from patients receiving auto-FMT compared to hetero-FMT. Pairwise comparisons were performed using Wilcoxon rank sum tests, with ns (not significant) for p>0.05, * for p <= 0.05, ** for p <= 0.01, *** for p <= 0.001, and **** for p <= 0.0001.


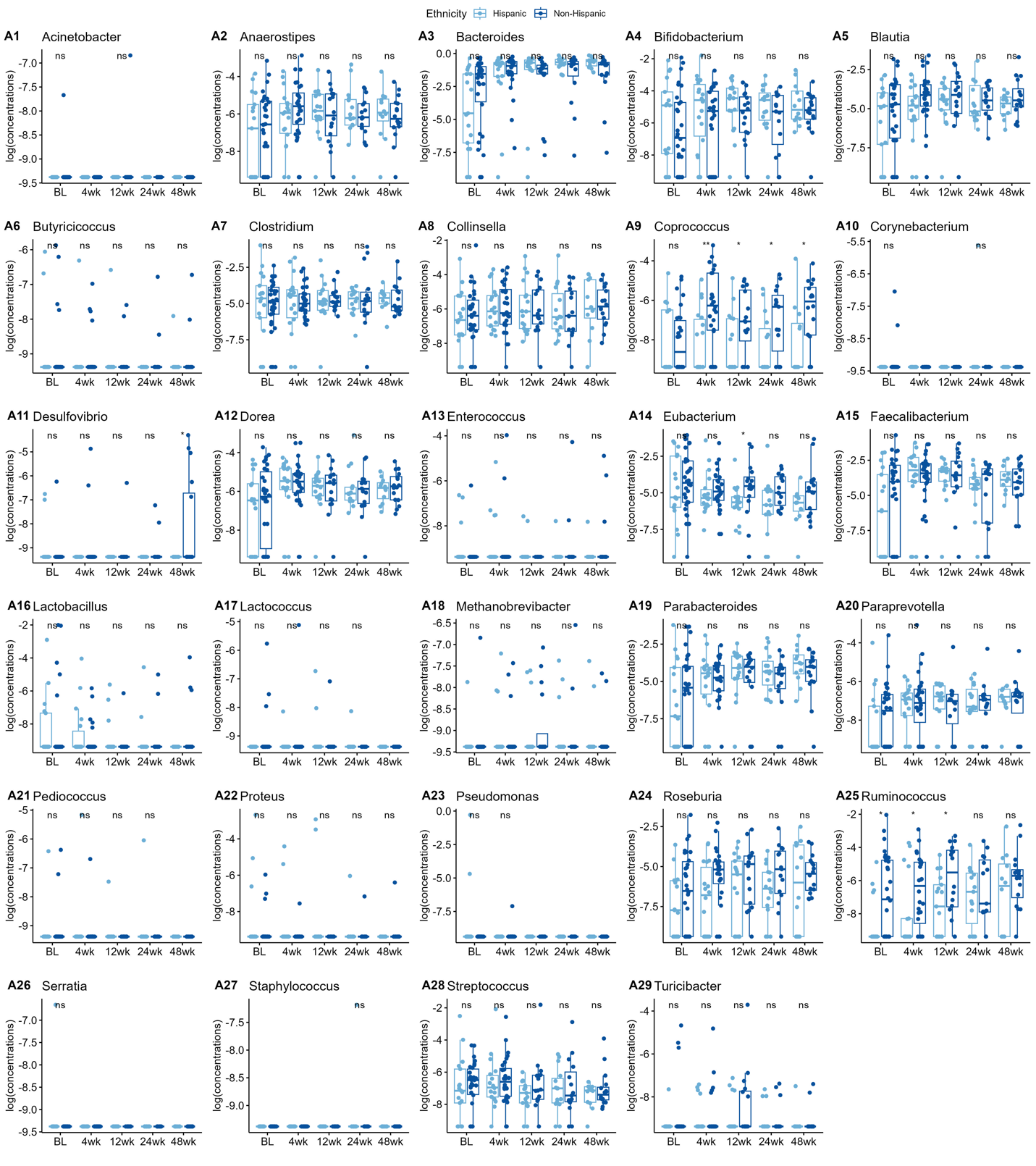


**Figure S11:** Boxplots of the log transformed relative abundance of different genus in different ethnicities. Pairwise comparisons were performed using Wilcoxon rank sum tests, with ns (not significant) for p>0.05, * for p <= 0.05, ** for p <= 0.01, *** for p <= 0.001, and **** for p <= 0.0001.


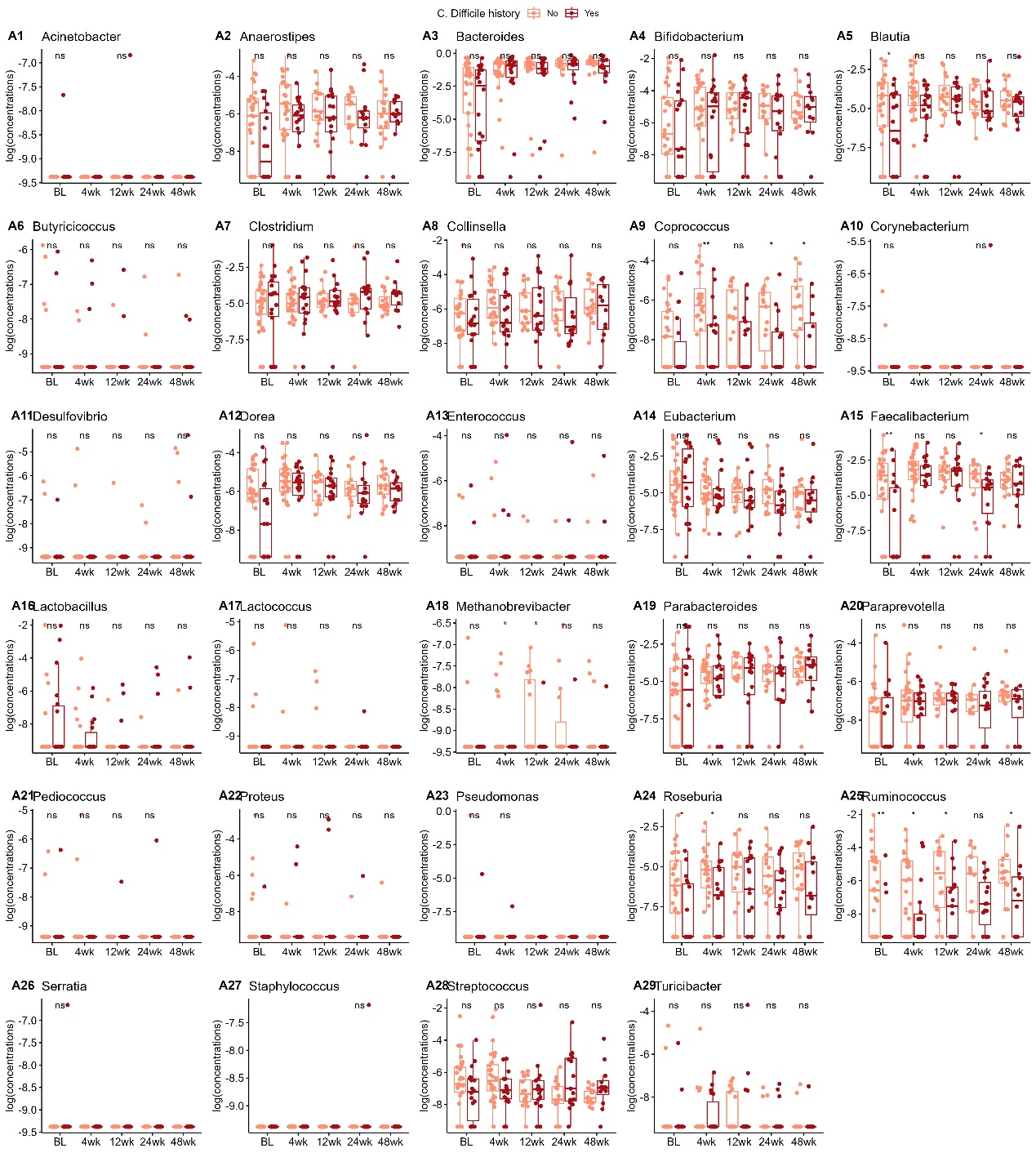


**Figure S12:** Boxplots of the log transformed relative abundance of different genera based on *C.difficle* history. Pairwise comparisons were performed using Wilcoxon rank sum tests, with ns (not significant) for p>0.05, * for p <= 0.05, ** for p <= 0.01, *** for p <= 0.001, and **** for p <= 0.0001.
